# A Statistical Framework for Modeling HLA-Dependent T Cell Response Data

**DOI:** 10.1371/journal.pcbi.0030188

**Published:** 2007-10-12

**Authors:** Jennifer Listgarten, Nicole Frahm, Carl Kadie, Christian Brander, David Heckerman

**Affiliations:** 1 Microsoft Research, Redmond, Washington, United States of America; 2 Partners AIDS Research Center, Massachusetts General Hospital, Charlestown, Massachusetts, United States of America; University of California San Diego, United States of America

## Abstract

The identification of T cell epitopes and their HLA (human leukocyte antigen) restrictions is important for applications such as the design of cellular vaccines for HIV. Traditional methods for such identification are costly and time-consuming. Recently, a more expeditious laboratory technique using ELISpot assays has been developed that allows for rapid screening of specific responses. However, this assay does not directly provide information concerning the HLA restriction of a response, a critical piece of information for vaccine design. Thus, we introduce, apply, and validate a statistical model for identifying HLA-restricted epitopes from ELISpot data. By looking at patterns across a broad range of donors, in conjunction with our statistical model, we can determine (probabilistically) which of the HLA alleles are likely to be responsible for the observed reactivities. Additionally, we can provide a good estimate of the number of false positives generated by our analysis (i.e*.*, the false discovery rate). This model allows us to learn about new HLA-restricted epitopes from ELISpot data in an efficient, cost-effective, and high-throughput manner. We applied our approach to data from donors infected with HIV and identified many potential new HLA restrictions. Among 134 such predictions, six were confirmed in the lab and the remainder could not be ruled as invalid. These results shed light on the extent of HLA class I promiscuity, which has significant implications for the understanding of HLA class I antigen presentation and vaccine development.

## Author Summary

At the core of the human adaptive immune response is the train-to-kill mechanism in which specialized immune cells are sensitized to recognize small peptides from foreign pathogens (e.g., HIV virus). Following this sensitization, these cells are then activated to kill other cells that display this same peptide (and that are infected by this same pathogen). However, for sensitization and killing to occur, the pathogen peptide must be “paired up” with one of the infected person's other specialized immune molecules—an HLA (human leukocyte antigen) molecule. The way in which pathogen peptides interact with these HLA molecules defines if and how an immune response will be generated, which has implications for vaccine design where one may artificially introduce select peptides to pre-train the immune system. Furthermore, there is a huge repertoire of such HLA molecules, with almost no two people having the same set. We introduce a statistical approach for identifying which HLA molecules interact with which pathogen peptides, given a particular kind of laboratory data. Our approach takes as input, data that tells us only which pathogen peptides generate a response, but not which HLA molecules support the response. Our statistical approach fills in this missing information.

## Introduction

The human adaptive immune response is composed of two core elements: antibody-mediated response (sometimes called humoral response), and T cell–mediated response (sometimes called cellular response). Research on HIV vaccines initially focused on the antibody-mediated response but more recently has included the cellular response [1,2], which is the focus of our application.

At the core of the cellular response is the ability of certain antigen-presenting cells to digest viral proteins into smaller peptides, and then to *present* these peptides at the surface of the cell. Presentation of a peptide depends on the peptide first forming a complex with an HLA (human leukocyte antigen) molecule. If a peptide is presented, it can then be recognized by (naive) T cells, allowing activation of these T cells so that they may subsequently recognize and attack virally infected cells displaying the same complex. Any peptide that is able to generate such an immune response in the context of a given HLA allele is called an *epitope*, and, in particular, an epitope *restricted by that allele*. Only certain HLA alleles can form a complex with any given peptide, and hence the compatibility of these two elements is essential for the adaptive immune response just described.

Several types of T cells exist, each playing its own, though interdependent, role. In ongoing HIV vaccine research, the elicitation of a CD8+ T cell response has shown promise. Since CD8+ T cells recognize only HLA class I bound epitopes, our data, and hence our paper, focus on epitopes recognized in the context of these particular molecules, although the statistical framework is not tailored or limited to this domain and could be immediately applied to HLA class II epitopes, for example. Humans have up to six HLA class I alleles arising from the A, B, and C loci. Currently, there are hundreds of possible alleles at each of these loci, with more being discovered every year [3].

A crucial task in HIV vaccine development is the identification of epitopes and the alleles that restrict them, since it is thought that a good vaccine will comprise a robust set of epitopes [4–6]. By robust, we mean a set which broadly covers regions that are essential for viral fitness in a given human population characterized by a particular distribution of HLA alleles. Also, note that beyond vaccine design, epitope identification may have important applications such as predicting infectious disease susceptibility and transplantation success.

Traditional methods for identifying epitopes involve time-consuming, technically demanding, and expensive culturing of T cells. Recently, a more expeditious laboratory technique using ELISpot assays has been developed [7]. Unfortunately, the ELISpot assay gives only information about which individual donors generated an immune response to a particular peptide, but does not provide any information about which of a donor's HLA alleles are restricting this reaction; it is this HLA specificity that is crucial and in which we are most interested. However, by leveraging information contained in ELISpot reactivity across a large set of donors with known HLA types, in conjunction with the statistical model presented in this paper, we can determine (probabilistically) which HLA alleles are likely to be responsible for the observed reactivities. Thus we are able to learn about new HLA-restricted epitopes in an efficient, cost-effective, and high-throughput manner.

A related, though distinct problem from our problem of epitope *identification* is that of epitope *prediction* (e.g., [8–11]), in which new epitopes are predicted in silico, on the basis of amino acid sequence and other information, but not on the basis of assays that directly measure binding energies or other measures such as the ELISpot assay. The work presented here focuses strictly on the identification of restricting (i.e., epitope presenting) HLA class I alleles from ELISpot data, although newly identified epitopes can aid the task of epitope prediction by providing more known examples to learn from.

## Methods

Our statistical model takes as input, measured CD8+ T cell ELISpot reactivities from a set of donors with known HLA class I alleles, for a number of epitopes, and deduces which of the donor's individual HLA alleles are likely to be responsible for the observed reactivities. That is, our model deduces which epitopes are restricted by which HLA alleles. Additionally, we can provide a good estimate of the number of false positive epitope hypotheses returned by our analysis (i.e., the false discovery rate (FDR) [12,13]) so that we have a sense of how many new epitope hypotheses to pursue (if any).

We assume that a given epitope is or is not restricted by a given HLA allele. If an epitope is restricted by a particular HLA allele, it is still likely that a donor with the restricting HLA allele will not react to the epitope. Such false negatives arise from factors including immunodominance, (immunodominance can be thought of as biology's “waste not want not”—that is, the immune system focuses its efforts in a few areas that work well, to the exclusion of others), T cell repertoire, lack of previous peptide exposure (e.g., exposure arising from infection or vaccination), suboptimality of the epitopes (i.e., if a peptide that optimally binds a particular HLA is of length nine amino acids, a peptide of length ten which contains the nine-mer may sometimes bind, but not as efficiently), and experimental noise. Furthermore, an epitope reaction may be falsely associated with some HLA alleles in ELIspot data due to linkage disequilibrium of a nonrestricting and a restricting HLA allele. (For example, if a restricting HLA allele is in linkage disequilibrium with a nonrestricting HLA allele, then the nonrestricting allele will very often be present in a donor with the restricting allele, and so the ELISpot data for this allele will also correlate with positive reactivities—though only as a result of the linkage.) Thus, the task of recovering HLA-restricted epitopes from ELISpot data is not straightforward. As a brief example, if one examines the HIV ELISpot dataset used in this paper and considers any HLA-epitope pair that has any observed reactivity to consist of an HLA-restricted epitope, then one incurs a false positive reactivity rate of roughly 70%. One could then imagine a next logical step of setting a threshold for what minimum fraction of donors must react and so on, soon finding oneself with a rather ad hoc model for which there would be no principled way to set the parameters nor to determine statistical significance. The task of identifying restricting HLA alleles from ELISpot data is in fact nontrivial and well-suited to statistical modeling. Next, we formally outline our statistical model.

### A Statistical Model for HLA-Dependent T cell Response Data

For a set of *J* epitopes (more precisely, each peptide under examination may contain one, or several, epitope(s), but for simplicity of presentation, we refer to the peptides as epitopes) and *K* donors, we have a set of measured binary ELISpot reactivities (actual laboratory assays provide real values which are thought by the laboratory scientists to convey mostly binary information [14]), which are used as input to our model. We are also given the six HLA class I alleles for each donor.

Let *h_i_* = 1 denote that a donor has HLA allele *i*, and *h_i_* = 0 denote that the donor does not have that allele. Let *y_j_* be the observed, binary reactivity for epitope *j* in a donor (as measured by the ELISpot assay). An important assumption in our model is the following: whether an epitope is restricted by a particular HLA allele is independent of whether that epitope is also restricted by any other HLA allele. This assumption is commonly referred to as an assumption of *causal independence* [15]. From this assumption, it follows that the probability of not observing a reaction to a particular epitope, in a given donor, is the probability that none of that donor's HLA alleles cause a reaction. Because of the independence assumption, this is simply the product over the probability of each HLA (that the donor has) not causing a reaction. Formally, if epitope *j* is restricted by HLA *i*, then we let *q_ij_* be the probability that we observe a reaction in a donor with HLA *i* and no other HLAs restricted by epitope *j*. Also, let *l_j_* be the probability that a reaction is observed to epitope *j* when a donor has none of the restricting HLAs for epitope *j*—a so-called *leak term* (corresponding to unrepresented causes such as reactivity due to HLA E molecules). Given settings for these parameters, *q_ij_* and *l_j_*, our model stipulates that the probability that a donor does not react to epitope *j*, *p*(*y_j_* = 0 | {*q_ij_*},*l_j_*), or does react, *p*(*y_j_* = 1 | {*q_ij_*},*l_j_*), is given by

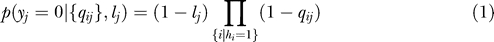


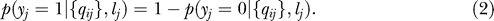



**Figure 1:**
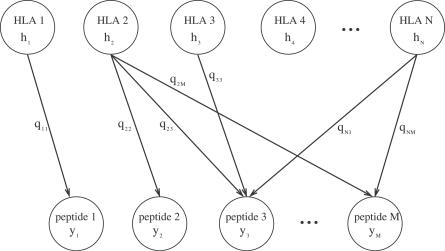
Graphical Depiction of HLA Restriction Model Graphical depiction of the model used to infer HLA-restricted epitopes from ELISpot data. The probability of each peptide having a reaction is parameterized by a noisy-OR distribution over all of the HLA alleles it is connected to (Equations 1 and 2). The values of the HLA and peptide nodes are observed for each donor, and we are interested in finding which *q_ij_* > 0—that is, which arcs are present in the graphical model. Each person has between three and six distinct HLA class 1alleles. Thus, for a given donor, between three and six HLA nodes will be “on” (*h_i_* = 1).

Such a model is sometimes referred to as a *noisy-OR model*. It can be viewed as a probabilistic version of the common (deterministic) logical OR, and has been shown to be useful in a number of settings [16]. The model can be represented in graphical form as shown in Figure 1. Here, nodes represent the variables {*h_i_*} and {*y_j_*} and an *arc* is drawn from *h_i_* to *y_j_* if epitope *j* is restricted by HLA *i* (i.e., if *q_ij_* > 0). The characteristics of how the probability of an observed reaction changes with an increasing number of restricting alleles depends on the values of {*q_ij_*}. For example, if *q_ij_* ≡ *q_j_ ≈* 1, then for a given donor with *M* restricting alleles, each additional restricting allele beyond one allele would do little to increase the probability of a reaction to epitope *j* (as with a deterministic logical OR). Alternatively, if *q_ij_* ≡ *q_j_ ≈* 0, then according to the Taylor series expansion 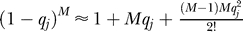 , the probability of reactivity to epitope *j* would increase roughly linearly with *M*. The likelihood of the ELISpot data under this model is simply the product of likelihood terms for the reaction in each patient k, to each peptide (given the HLA types for each patient) :

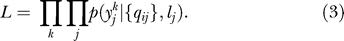



### Finding HLA-Restricted Epitopes

Given the model just described, and experimental ELISpot and donor HLA data, we wish to infer which epitopes are restricted by which HLA alleles. That is, we wish to know which *q_ij_* should be included in the model (which arcs should appear in the graphical model). This is a problem of *model selection*. Note that this problem breaks down into *J* separate problems, one for each epitope under consideration, since under our model, *q_ij_* and *q_i′j′_* are independent from one another when *j* ≠ *j′*.

To tackle this problem of inferring HLA-restricted epitopes from our data and model, one might consider simply learning a maximum likelihood value for all possible *q_ij_* simultaneously, and concluding that those for which *q_ij_* > 0 are those which support the hypothesis of an HLA-restricted epitope. However, in practice, with finite and noisy-data, almost all *q_ij_* > 0, and this approach would lead to a huge number of false epitope hypotheses. Instead, we need a more robust way of deciding which *q_ij_* to include in the model. There are a variety of standard approaches to this problem, most centered on some form of *model selection score*, such as the Akaike Information Criterion (AIC) [17], the BIC (Bayesian Information Criterion) [18], or the MDL (Minimum Description Length) [19]—all of which are forms of *penalized likelihood* scores. These scores are but three commonly used model selection scores, and many variations of these exist as well. However, all of these scores have an intuitive interpretation of balancing the fit of the data to the model, with model complexity (controlling the model complexity so that overfitting does not occur). The fit of the data to the model is usually assessed by the maximum likelihood of the data under the model in question, while the model complexity is usually controlled by penalizing for the number of free parameters in the model—hence the term penalized likelihood. For example, the AIC of a model, *M*, is given by 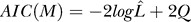 , where 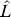 is the maximum likelihood, and *Q* is the number of independently adjusted parameters in the model.

Given a model selection score, one then chooses a search procedure to select *q_ij_* (arcs) for inclusion in the model. The ideal way to do so is to try every subset of arcs and choose the subset which gives the highest model score (for example). However, with *n* possible arcs per epitope there are 2*^n^* subsets, and this approach is not feasible for most problems. Thus, in practice, it is common for some form of greedy, stepwise procedure to be used, such as greedily adding arcs to the model, or greedily adding/deleting arcs, terminating when the model score can no longer be increased. Then the final model built in the greedy sequence of models is taken as the model to be used and/or interpreted. Commonly, the search is started with the empty model (no arcs). In synthetic experiments with our model, we found that a greedy add/delete procedure, starting from the empty set, worked well (see Results for details), and thus we use such a procedure to identify specific HLA alleles restricting given epitopes.

It may at first seem counterintuitive that deleting an arc could increase the score when in a previous step adding that same arc had increased the score. However, when one considers that different variables can explain the same data to differing degrees, then it becomes clear how this can arise. Suppose one arc most explains some part of the data, followed next by, say, two other arcs, each of which explains that part of the data less well than the first arc, but which together explain the data better than the first arc by itself. In this case, after addition of the first arc, followed by addition of the next two arcs, the first arc would become redundant in light of the other arcs, and so removing it can increase the model selection score (it will not improve the likelihood, but will incur a parameter penalty). In practice, for our problem and data, the delete operator was used only occasionally.

Different model selection scores used in a given search procedure lead to different recovered models. In particular, AIC is known to be generally less conservative (allowing more arcs) as compared with, say, BIC and MDL. Note that if one were to use an add-only procedure (where deletion of an arc is not allowed) for noisy-OR based models, then the AIC, BIC, MDL, and the Likelihood Ratio Test (LRT) [20] would each add arcs in the same greedy order, though with each score stopping at a different point in the search (except for BIC and MDL which are equivalent). So the fundamental difference between these scores is not so much which arc to add next, but when to stop adding arcs.

Rather than dogmatically choosing one score with which to find restricting HLA alleles, we develop a novel approach in which we use a parameterized *family of model scores*. Then, for any chosen model score parameter setting, we are able to estimate the FDR of the resulting model (that is, we are able to estimate the proportion of recovered *q_ij_* which are not truly HLA-restricted epitopes). Then we choose a model score parameter setting which produces an FDR that we find reasonable for our purposes (i.e., one producing an FDR that gives us enough epitope hypotheses to pursue, but not too many false leads). This approach to model selection confers two advantages over the more traditional approach described: (1) we do not depend in a fundamental way on the choice of a single model selection score, and (2) regardless of which model selection score we use (within the parameterized family), we are able to estimate the FDR of our selected arcs, providing us with a good sense of what (interpretable) features the model has actually recovered, rather than, say, far less interpretable measures of quality such as the maximum likelihood of the data under the recovered model compared with that under some baseline model.

### A Parameterized Family of Model Selection Scores

We call the parameterized family of model scores XIC, (to denote that it encompasses various Information Criterion such as AIC and BIC). The XIC for model *M* is parameterized by *f* and is given by

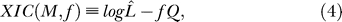
where 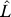 is the maximum likelihood of model *M* (*M* represents, for example, a model consisting of a particular subset of {*q_ij_*}), *Q* is the number of independently adjusted parameters in the model, and *f* parameterizes the family of scores represented by XIC. When *f* = 1, the XIC behaves identically to the (negative) AIC during search, because it is directly proportional to it. When *f* = ½ log *N*, where *N* is the sample size of the data, then the XIC is identical to the BIC. When *f* = 0, the XIC is the maximum likelihood. Thus by varying *f*, the XIC spans a range of model selection scores, from very liberal ones for low values of *f*, to increasingly conservative ones for higher values of *f*.

### Model Selection Procedure

Leaving aside the issue of estimating the FDR for the moment, our model selection procedure is the following:

1. Select a value for the XIC parameter, *f* = f^*^.

2. Start with the empty set of arcs under consideration (that is, no *q_ij_* are in the initial model), but include all of the leak terms, *l_j_*. Compute the XIC of this “leak-only” base model, *M*_0_.

3. For every *q_ij_* under consideration, compute the XIC of the model which is the same as *M*_0_ but also includes *q_ij_*. If none of these models has a higher XIC than *M*_0_, stop the search. Otherwise, add the *q_ij_* whose corresponding XIC was largest, and call the resulting model, *M*_1_.

4. Repeat the previous step, except using *M*_1_ in place of *M*_0_, and also allowing arc deletions: for all *q_ij_* in *M*_1_, compute the XIC of the model which is the same as *M*_1_, except that it *does not contain q_ij_*. Among all the possible arc additions and deletions, choose the operation which most increases the XIC, and call the resulting model *M*_2_.

5. If possible, continue greedily adding/deleting arcs, stopping when the XIC can no longer be increased.

Then we use the last model in the sequence as our final model from which to infer HLA-restricted epitopes. That is, for all *q_ij_* included in the final model, we will call the hypothesis that epitope *j* is restricted by HLA *i*, true. The smaller f^*^ is, the more *q_ij_* will be included in the final model. Next we show how to estimate the number of *q_ij_* recovered using this procedure that we expect to be spurious (i.e., arising from chance alone, rather than from true HLA restrictions).

### Estimating the False Discovery Rate

For any specified value of the model selection parameter, *f*, we want to know how many *q_ij_* in the recovered model are likely to be true (rather than spuriously generated). That is, we want some sort of statistical significance measure for the epitope hypotheses we have generated. We compute such a measure using a method that we have recently developed [21]. Next we provide some background to this area of research, followed by presentation of our approach.

When inferring whether a single hypothesis is true or not, statisticians have traditionally relied on the *p*-value, which controls the number of false positives (type I errors). However, when testing hundreds or thousands of hypotheses simultaneously, the *p*-value needs to be corrected to help avoid making conclusions based on chance alone (known as the problem of *multiple hypothesis testing*). A widely used, though conservative correction, is the Bonferroni correction, which controls the Family Wise Error Rate (FWER). The FWER is a compound measure of error, defined as the probability of seeing at least one false positive among all hypotheses tested. In light of the conservative nature of methods which control the FWER, the statistics community now places great emphasis on estimating and controlling a different compound measure of error, the *false discovery rate* (FDR) [12,13].

In a typical computation of FDR, we are given a set of hypotheses where each hypothesis, *i*, is assigned a score, *s_i_* (traditionally, a test statistic, or the *p*-value resulting from such a test statistic). The FDR is computed as a function of a threshold, *t*, on these scores, *FDR = FDR*(*t*). For threshold *t*, all hypotheses with *s_i_* ≥ *t* are said to be significant (assuming, without loss of generality, that the higher a score, the more we believe a hypothesis). The FDR at threshold *t* is then given by

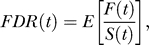
where *S*(*t*) is the number of hypotheses deemed significant at threshold *t* and *F*(*t*) is the number of those hypotheses which are false, and where expectation is taken with respect to datasets of the same sample size as the observed data drawn from the true joint distribution of the variables. When the number of hypotheses is large, as is usually the case, one can take the expectation of the numerator and denominator separately:

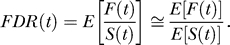



Furthermore, it is often sufficient to use the observed *S*(*t*) as an approximation for *E*[*S*(*t*)]. Thus, the computation of *FDR*(*t*) boils down to the computation of *E*[*F*(*t*)]. One approximation for this quantity which can be reasonable is *E*[*F*(*t*)] ≅ *E*_0_[*F*(*t*)], where *E*_0_ denotes expectation with respect to the null distribution (the distribution of scores obtained when no hypotheses are truly significant), and it is this approach that we take. (For traditional applications of FDR, Storey and Tibshirani offer a clever method to compute *E*[*F*(*t*)] which is less conservative than using *E*[*F*(*t*)] ≅ *E*_0_[*F*(*t*)] [13]. However, this approach is not appropriate in the present context.)

Applying this approach to estimating the number of true *q_ij_* recovered by our model selection procedure (i.e*.*, the number of true HLA-restricted epitopes found by our model), we generalize *S*(·) and *F*(·) to be functions of *f*, the XIC parameter in Equation 4. In particular, *S*(*f*) is the number of *q_ij_* found by our model selection procedure when the XIC is used with parameter setting *f* and *F*(*f*) is the number of those *q_ij_* which do not truly correspond to HLA-restricted epitopes (i.e., false positives). As in the standard FDR approach, we use the approximation *E*(*S*(*f*)) ≅ Q(*D*,*f*), where *Q*(*D*,*f*) is the number of *q_ij_* found by applying our model selection procedure with XIC parameter *f* to the observed data *D* (in our application, *D ≡* {*y_j_*}). In addition, we estimate *E*_0_(*F*(*f*)) to be *N*(*D^r^*,*f*) averaged over multiple datasets *D^r^*, *r* = 1,…,*R*, drawn from a null distribution. That is, we estimate the FDR of our HLA-restricted epitopes using the following:

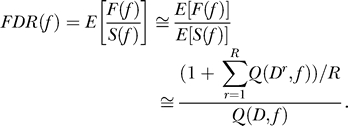



The addition of 1 to the numerator smoothes the estimate of *E*_0_[*F*(*f*)] so as to take into account the number of random permutations performed. Without this smoothing, if one performed too few random permutations such that ∑*_f_Q*(*D^r^*,*f*) = 0 due to sampling error, then the estimate of *E*_0_[*F*(*f*)] and hence FDR (*f*) would also be 0. We prefer our more conservative estimate, especially as the bias it induces diminishes as the number of permutations increases.

We sample *D^r^* from a null distribution for each epitope by permuting the ELISpot data for that epitope, but leaving the HLA types of the donors intact. This permutation guarantees that any *q_ij_* recovered from the model selection procedure on this data are only spuriously recovered. Also note that although the parameters *q_ij_* are independent for different epitopes, *j*, and thus the model selection procedure, can operate independently on each epitope, for the purposes of estimating the FDR, we pool all of the epitopes together, so that the approximations we make in computing the FDR are more reasonable.

As shown in the Results section, by way of synthetic experiments, we find that these approximations for estimating the FDR work quite well in practice. There is, however, one concern about the use of the null distribution described, for which we refer the reader to [21], but which, to our knowledge, does not affect our use of this methodology in this paper.

By construction, the emphasis of our FDR approach is on the accuracy of the estimate of the number of false positives, and does not examine the number of false negatives. Whereas this emphasis may seem undesirable, it is common for experimenters to be more interested in how many hypothesized interactions are real, rather than how many were missed, because experimenters will in most cases be using resources to pursue the positive hypotheses, not the negative ones. A similar line of reasoning is mentioned in [13,22].

### Ranking of Hypotheses

The problem of finding a meaningful ranking of the individual HLA-restricted epitope hypotheses does not immediately fall out of the FDR framework. However, we can naturally construct a ranking algorithm for the epitope hypotheses by using a Likelihood Ratio statistic. Let *M* denote the model that we learn with our model selection procedure (regardless of the value of *f* used). Then we rank our hypotheses using a likelihood ratio statistic, *v_ij_*, which is the log of the ratio of the likelihood of the final model, to that of the final model without the *q_ij_* we are evaluating. Specifically, our ranking algorithm is:

For each *q_ij_* included in *M*, do the following: construct a model, *M′_ij_* defined to be model *M*, but without *q_ij_*, and then compute the likelihood ratio:

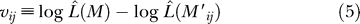



Assign a rank to each *q_ij_* equal to the rank of *v_ij_* in the set {*v_ij_*}.

This ranking assesses each *q_ij_* based on how much it contributes to the likelihood of the data in the model, *M*, in the context of all *q_ij_* recovered from the model selection procedure. (The likelihood ratio, *v_ij_*, viewed from a Bayesian perspective, is a quantity proportional to a BIC approximation to the Bayes factor [18], which, under the assumption of a uniform prior over arc sets, amounts to the posterior probability of *q_ij_* being included in the model, given the remaining arcs in the model.)

## Results

For our experiments, we used two types of datasets: laboratory-generated HIV ELISpot data, as well as synthetic data based on our model and this real data. The HIV ELISpot data is from a set of previously optimally defined CTL epitopes derived from HIV [14], which were generally optimized for length so as to be recognized at the lowest antigen concentration in the context of a specific restricting HLA class I allele. Note that these “optimal” peptides may not be optimal for other HLA class I alleles which could also restrict them—for example, other alleles could restrict epitopes that are embedded within the longer peptide sequence tested. There were 140 epitopes and 102 donors with a total of 70 unique HLA-I alleles (although HLA alleles are ideally described by a four-digit number; in many cases, this was not available, and as such, we truncated all HLA-I alleles to two digits, with the exception of the HLA-B15 family alleles, which always had the full four digits available since these “subtypes” may present vastly different sets of epitopes [23,24]. The number of unique HLA alleles reported is the number obtained after this compression.). First we use synthetic experiments to show that (1) the FDR estimate we have described is reasonably accurate, and (2) the model selection procedure can recover a good proportion of ground-truth HLA-restricted epitopes from data. Finally, we apply our algorithm to the real data.

Note that to compute the XIC score for our models, we need to find the maximum likelihood solution for noisy-OR nodes. Fortunately, this is a convex optimization problem [25] and therefore local minima are not a problem.

### Synthetic Experiments

The synthetic model used to generate data was our epitope model, as described earlier, fitted to the real HIV ELISpot data by using our model selection procedure. We used an XIC setting for *f* that resulted in an estimated *FDR* ≅ 0.3 (*f* = 2.9). This produced 165 *q_ij_* in our synthetic model. Additionally, we retained the learned maximum likelihood values for these *q_ij_* (and the leaks, *l_j_*), so as to be able to generate data from the model. To generate synthetic data from this fitted model, donor HLA data was left as it appeared in the real data, and then Equations 1 and 2 were used to compute the probability that a particular donor would react to a particular epitope, *p_j_*, conditioned on the learned values of {*q_ij_*} and {*l_j_*}. Then samples for each donor, *s_j_*, were drawn from a uniform distribution on (0,1] and the reactivities, *y_j_*, were set to *y_j_* = ( *s_j_* ≤ *p_j_*). Three synthetic datasets (each consisting of 102 donors and 140 epitopes) were generated in this manner, all from the same synthetic, generative model.

**Figure 2:**
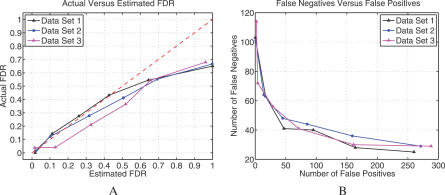
Actual versus Estimated FDR (A) and False Negatives versus False Positives (B) Results from using our model selection procedure and FDR estimation procedure on three datasets generated from a synthetic model learned on the real HIV data. There is a one-to-one correspondence between the points plotted in each figure. (A) Estimated and actual FDR. The dashed line denotes the idealized curve. (B) The number of false negatives (*q_ij_* not recovered in these experiments, but appearing in the synthetic model), compared with the number of false positives (*q_ij_* recovered in these experiments, but not in the synthetic model).

Plots of actual versus expected FDR for the three datasets are shown in Figure 2A. Estimates of FDR are quite accurate at the lower end, which is the region of interest for our problem and also most other problems of interest (where not too many spurious hypotheses are included). That the FDR becomes increasingly conservative (i.e*.*, it peels away from the idealized line) can likely be explained by the approximation we make in generating a null distribution. Further discussion of this issue, and a suggested resolution, can be found in [21]. For the XIC parameter, *f*, we used the range [1.97,3.46], with *f* = 3.46 producing actual and estimated FDRs around 0.02, and *f* = 1.97 producing actual FDRs around 0.67 and estimated FDRs around 0.95. Note that BIC corresponds to the cluster of points that have estimated FDRs around 0.7 (*f* = 2.3). AIC (*f* = 1) corresponds to something even less conservative than anything shown (even higher FDRs).

Not only do we want to know that our FDR estimate is accurate, but we also want to know that our model selection procedure is a reasonable one. We therefore examine how many ground truth *q_ij_* were recovered, and at what cost in false negatives. This information is displayed in Figure 2B. Note that because we created a synthetic model with what were presumed to be 30% spurious *q_ij_*, many of these *q_ij_* are likely quite small (signifying weak associations), and therefore would be more difficult to recover in synthetic experiments using data generated from this model. Such difficulties are also likely to arise with real data in real applications. The points in Figure 2B that have about 50 false positives correspond to an estimated/actual FDR of around 0.3. The points which have about 150 false positives are those corresponding to *XIC* = *BIC* (for which *FDR* ≅ 0.7). Overall, the tradeoff between the number of false positives and false negatives is very reasonable.

### Application to Real Data

Using the real HIV data, we found 134 HLA-restrictions at *FDR* ≅ 0.2 among the possible 140 × 70 possible HLA restrictions. To validate our predictions on the real HIV dataset, we performed in vitro assays that specifically measured particular HLA restrictions [26]. Ideally, all 134 pairs should have been evaluated, but this was too expensive and work-intensive. Consequently, six pairs for which the HLA-peptide association is biologically interesting (i.e*.*, unlikely based on current understanding of peptide–HLA binding) were evaluated. All six relationships were confirmed [26]. Prior to this study (partially reported in [26]), it was thought that HLA class I epitopes were restricted mainly by a single HLA allele, or if by more than one allele, then only a few that were structurally highly related and commonly fell into the same HLA supertype [27] (supertypes group together HLA alleles with similar amino acid binding motifs). However, our analysis suggests that a single epitope is frequently restricted by numerous HLA alleles. Additionally, when viewed through the traditional lens of supertypes, we found restrictions across supertypes. For example, IYQEPFKNLK was previously known to be restricted by A11, and we found that it is also restricted by A24 (confirmed experimentally), where A11 and A24 belong to two different supertypes. Table 1 shows a summary of the number of previously known HIV epitopes restricted by one HLA allele, and up to four HLA alleles (none were known to be restricted by more than four alleles) [14]. After adding our newly statistically identified HLA-restricted epitopes, these numbers change dramatically, as shown in Table 2. These tables suggest that HLA class I epitopes are far more “promiscuous” than originally thought, a notion that has significant implications for the understanding of HLA class I antigen presentation and vaccine development. We refer the reader to [26] for a more detailed account of the biological findings. (Note that there are a few differences between the results reported in [26] and the current presentation of results. In [26], the previously known HLA-restrictions were “fixed” to be present in the model before model selection was used to search for new HLA restrictions. We thought it would be of interest to see the results when this a priori information was not used. Additionally, the number of HIV “optimal” epitopes tested was reported as 162 in [26], whereas we report 140—this is due to the fact that epitope–HLA pairs were counted in the former, while here we count only unique epitopes—of which some were repeated across HLA restrictions. The raw data are, however, identical.)

**Table 1:**
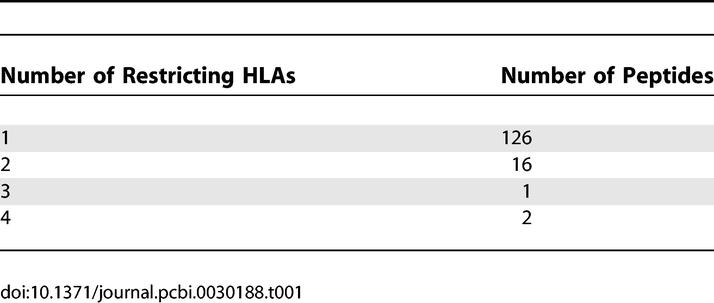
Previously Known Promiscuity

**Table 2:**
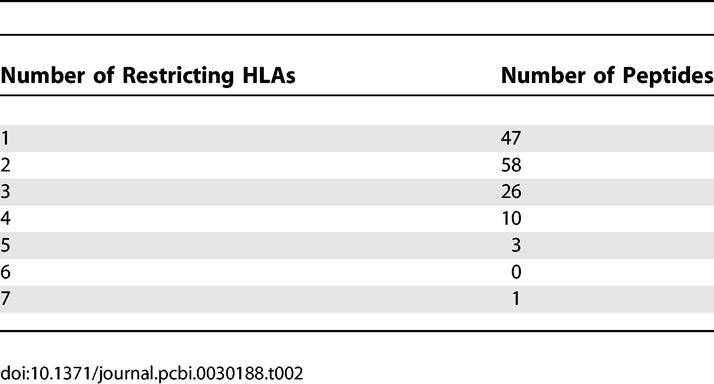
Promiscuity Updated with Present Analysis

Table S1A lists all epitopes identified by our statistical analysis, sorted by rank from most to least important, along with their learned *q_ij_* values, and noting which epitopes were previously known, which were confirmed, and what other HLA alleles were previously known to restrict each epitope. Of the 134 identified epitopes we identified, 46 were previously known (eight of our top ten ranked epitopes were known).

## Discussion

We have introduced, implemented, and examined use of a statistical approach for identifying epitope-restricting HLA alleles from ELISpot data. This approach provides a high-throughput, efficient, and cost-effective method for the screening of novel HLA-restricted epitopes. Additionally, our methodology introduces a new approach to the model selection problem, wherein a parameterized family of model selection scores can be explored, by estimating the FDR resulting from the use of each score, and choosing one which suits the needs of the user. In other words, we are able to customize the tradeoff between high discovery rates, and false leads, rather than relying on a single model selection criterion.

Several improvements to the model are possible. (1) Some donors tend to have a higher overall reaction level, thus it may be fruitful to include a latent variable which models this donor-specific bias. (2) A confounding factor in our analysis is the existence of false negatives due to a failed chemical reaction in the ELISpot assay. One could add an observation component to model this type of experimental noise. (3) We stated that the ELISpot data are real-valued, but thought to be informative at a mostly binary level. However, it might be possible to extract more information by using the actual real-valued measurements.

Lastly, by applying our methodology to real HIV data, we have helped to shed light on the extent to which HLA class I epitopes are promiscuous. This has significant implications for the understanding of HLA class I antigen presentation and vaccine development.

## Supporting Information

Table S1 Comprehensive List of All HLA-Restricted Epitopes Found on HIV Data Known HLA refers to HLA restrictions previously known.
Recovered HLA refers to restrictions recovered from our statistical analysis.
*v_ij_* is the likelihood ration score used to rank the hypotheses (they are shown ranked from strongest to weakest).
*q_ij_* is the learned value of the noisy-OR parameter for HLA restriction.
Known is equal to one if this HLA restriction was already known.
Tested refers to those peptides which we confirmed experimentally.
(37 KB XLS)

## Abbreviations

AIC: Akaike Information Criterion
BIC: Bayesian Information Criterion
FDR: false discovery rate
FWER: Family-Wise Error Rate
HLA: human leukocyte antigen
MDL: Minimum Description Length
